# Menstrual blood-derived stromal cells: insights into their secretome in acute hypoxia conditions

**DOI:** 10.1186/s10020-023-00646-1

**Published:** 2023-04-04

**Authors:** María Ángeles de Pedro, María Pulido, Verónica Álvarez, Federica Marinaro, Ana María Marchena, Francisco Miguel Sánchez-Margallo, Javier G. Casado, Esther López

**Affiliations:** 1 Stem Cell Therapy Unit, Jesús Usón Minimally Invasive Surgery Centre, 10071 Cáceres, Spain; 2 RICORS-TERAV Network, ISCIII, 28029 Madrid, Spain; 3 Immunology Unit, University of Extremadura, 10003 Cáceres, Spain; 4 Institute of Molecular Pathology Biomarkers, University of Extremadura, 10003 Cáceres, Spain

**Keywords:** Menstrual blood, Mesenchymal stromal cells, Secretome, Acute hypoxia, Wound healing, Stem cell Therapy

## Abstract

**Background:**

Despite constant advances in regenerative medicine, the closure of chronic wounds is still challenging. Therapeutic approaches using locally administered MSCs have been considered a promising option. However, the viability of these cells is seriously threatened by acute hypoxic stress linked to wound healing. In this work, we aimed to study the tolerance of Menstrual blood-derived stromal cells (MenSCs) to acute hypoxia and their therapeutic paracrine effect.

**Methods:**

Isolated MenSCs were phenotypically characterized and evaluated in terms of proliferation, viability, and gene expression, under acute hypoxia (AH) compared with conventional cultured condition or normoxia (N). A step further, the secretome of MenSCs under acute hypoxia was analyzed with respect to their miRNAs content and by in vitro functional assays. For the analysis of differences between the two groups, Student’s *t*-test was performed and one-way ANOVA and Tukey’s multiple comparisons test for multiple groups were used.

**Results:**

Our results revealed that the viability of MenSCs was not affected under acute hypoxia, although proliferation rate slowed down. Gene analysis revealed 5 up-regulated (*BNIP3*, *ANGPTL4*, *IL6*, *IL1B*, and *PDK1*) and 4 down-regulated genes (*IDO1*, *HMOX1*, *ANGPTL2*, and *HGF*) in AH compared to N. Global gene expression analysis revealed a decrease in the gene ontology functions of migration and wound response with respect to the normoxic condition. In contrast, functions such as angiogenesis were enriched under the AH condition. Regarding the secretome analysis, two miRNAs involved in angiogenic processes (hsa-miR-148a-3p and hsa-miR-378a-3p), were significantly up-expressed when compared to the normoxic condition, being *MYC* gene, the unique target of both. Functional assays on HUVECs revealed a potential pro-angiogenic capacity of MenSCs cultured in both oxygen conditions (N and AH) based on the wound closure and tube formation results of their released paracrine factors. However, when compared to normoxia, the paracrine factors of MenSCs under acute hypoxia slightly reduced the proliferation, migration, and in vitro wound closure of HUVECs.

**Conclusions:**

MenSC exhibited a good survival capacity under acute hypoxic conditions as well as beneficial properties applicable in the field of tissue regeneration through their secretome, which makes them a potential cell source for wound healing interventions.

**Supplementary Information:**

The online version contains supplementary material available at 10.1186/s10020-023-00646-1.

## Introduction/background

Menstrual blood is an important source of mesenchymal stromal cells (MSCs), referred to as Menstrual blood-derived stromal cells (MenSCs). Their clinical potential is attracting widespread interest due to their high proliferative rate, periodic acquisition in a non-invasive manner, low immunogenicity, and lack of ethical issues when compared with other sources of MSCs (Chen et al. 2019b, 2021). MenSCs could be stably expanded for at least 20 passages without mutations or visible abnormalities in vitro and their lifespan is relatively long in comparison with other MSCs (Bozorgmehr et al. 2020). Moreover, MenSC proliferative capacity does not appear donor-dependent in individuals aged up to 40 years (Chen et al. 2015). Their administration has proved to be safe in acute and chronic tumorigenicity/toxicity experiments in immunocompromised mice (Bockeria et al. 2013). For all these considerations, MenSC safety has been proved, regardless of the donor of origin, or the cell passage. However, small modifications in the surface markers of representative of the MenSCs phenotype are seen as the passage number increases (Chen et al. 2015).

In addition to the qualities mentioned above, MenSCs have other properties that increase their potential in the field of regenerative medicine, such as the ability to migrate into the injury site, regulate the immune response, and promote the repair of damaged tissue. Additionally, they stimulate functional recovery, improving tissue regeneration and protecting target cells from apoptosis or further injury (Chen et al. 2019b, 2021).

On the other hand, despite constant advances in wound healing, traditional treatment approaches are insufficient, especially for patients with comorbidities, such as diabetes. Among the several factors contributing to the non-healing of wounds, reduced angiogenesis, and dysregulation in cytokine production by immune cells and local fibroblasts play a key role (Cuenca et al. 2018). In this sense, new therapeutical approaches using MSCs have been considered promising in tissue regeneration. However, the efficacy of MSCs is limited due to the poor survival rate after transplantation (Sylakowski et al. 2020). Many studies have shown that MSCs are able to tolerate moderate hypoxia, but not the severe hypoxia that predominates in many diseases involving injured tissue (Sylakowski et al. 2020; Khasawneh et al. 2019). Under physiological conditions, MSCs can experience low O_2_ concentrations ranging from 1 to 15% in their niche. For example, bone marrow MSCs reside at 1–7%, while adipose MSCs reside at 10–15% (Samal et al. 2021). For MenSCs, the physiological oxygen concentration in the human uterus is 2% (Brouillet et al. 2021). Endometrial hypoxia has been confirmed during menstruation in mouse models (Reavey et al. 2021), macaque models, and ex vivo human endometrial studies (Martínez-Aguilar et al. 2021). However, these oxygen concentrations are far from the concentration that has been measured in the wound bed (less than 1%) (Antebi et al. 2018).

One of the reasons that most MSCs are not able to survive the hypoxic environment within injured tissue is that they normally expand under atmospheric oxygen tension, or normoxia (20.9%) (Antebi et al. 2018). In this regard, it would be interesting to evaluate the behavior of MenSCs in the microenvironment of nonhealing wounds through the simulation of an acute hypoxic environment.

Hypoxic preconditioning is frequently used to potentiate the therapeutic effect of MSCs. Hypoxia culture not only reinforces the stemness of MSCs but also modifies the gene expression patterns, improving angiogenic and regenerative functions of the hypoxia-cultured cells, among others (Lee and Kang 2020; Noronha, et al. 2019). Acute Hypoxia culture (< 1% oxygen concentration) may prepare the cells for a similar environment to which they will be exposed when administered into injured tissue. This preconditioning modifies the phenotype of MenSCs and influences secretome production, in terms of soluble factors, as well as extracellular vesicles (EVs) (Almeria et al. 2019; Yang et al. 2022). The therapeutic potential of MenSCs (Chen et al. 2019a) and their secretome (Chen et al. 2021) has been evaluated in several diseases, however, to our knowledge, the effect of acute hypoxia on in vitro cultured MenSCs and their secretome have not been studied yet.

Therefore, the aim of this study was twofold: to characterize the effect of acute hypoxia on in vitro cultured MenSCs and to evaluate the therapeutic potential of their secretomes focusing on their applicability in wound closure. Our results revealed that many of the properties of MenSCs, under acute hypoxia conditions, were maintained. Interestingly, some of them, such as MenSC immunomodulatory potential and homing, were enhanced. Moreover, MenSC-derived secretomes could promote proliferation, migration, wound closure, and tube formation in Human Umbilical Vein Endothelial Cells (HUVECs). These findings make MenSCs considered an excellent candidate for regenerative therapy.

## Methods

### Isolation and culture of MenSCs

All experimental procedures were approved by the Ethics Committee of the Jesús Usón Minimally Invasive Surgery Center and written informed consent was obtained from each donor. Five healthy premenopausal women, aged between 26 and 41 years, with regular cycles, participated without any type of hormonal treatment as menstrual blood donors. MenSCs were isolated from human menstrual blood samples and characterized, as previously described (Pedro et al. 2021). MenSCs at passages P4-P6 were cultured in T175 flasks (Thermo Fisher Scientific, MA, USA) at a density of 80%. Dulbecco’s Modified Eagle’s medium (DMEM) supplemented with 10% fetal bovine serum (FBS) (Gibco, Thermo Fisher Scientific, Bremen, Germany), 1% penicillin/streptomycin, and 1% glutamine, was replaced every 3 days. For normoxia, MenSCs were cultured under standard conditions (37 °C with 5% CO_2_) and for acute hypoxia (0.1–1% O_2_, 5% CO_2_, 37 °C).

### MenSC surface marker expression

The guidelines of the International Society for Cell Therapy (ISCT) (Galipeau et al. 2016) were followed to confirm the purity and the identity of the isolated stromal cells. The expression level of positive cell surface markers (CD29, CD44, CD73, CD90, and CD105) and negative markers (CD14, CD34, CD45, and HLA-DR-), and their in vitro differentiation capacity towards osteoblasts, adipocytes, and chondroblasts, were determined as previously described (Pedro et al. 2021). Afterward, MenSCs under Normoxia (N) and Acute Hypoxia (AH) were analyzed by flow cytometry for the expression of 24 markers associated with immune response, apoptosis, angiogenesis, and cell adhesion and migration (Additional file 1: Table S1). Briefly, five primary MenSC culture lines per each condition at passages P4–P8 were incubated with different antibodies for 30 min at 4 ºC in PBS with 2% of FBS. After washing, cells were resuspended in PBS and acquired in a FACSCalibur™ cytometer (BD Biosciences, CA, USA) equipped with CellQuest software (BD Biosciences). The phenotypic profiles were compared in terms of the Mean Fluorescence Intensity (MFI).

### MenSC proliferation and viability assays

The same five primary MenSC lines, at passages P6–P9, were seeded at 2.0 × 10^4^ cells/wells in 24-well plates (Thermo Fisher Scientific) under the two above-mentioned culture conditions (N, and AH). Proliferation was evaluated quantitatively by Cell Counting Kit 8 (CCK-8) (Boster Bio, CA, USA), according to the manufacturer´s instructions, and qualitatively by image using an inverted microscope (Nikon Elipse TE2000-S) to 4× and 10×. Both analyses were performed at 0, 24, 48, and 72 h. For viability assessment, MenSCs were stained at 72 h with a fluorescent Live/Dead Cell Viability Kit, in accordance with the instructions of the manufacturer (Invitrogen, Thermo Fisher, MA, USA). All experiments were performed in triplicate.

### MenSC gene expression

To determine MenSC gene expression in the experimental conditions (N and AH), a panel of 60 genes associated with hypoxia or/and angiogenesis was evaluated by real-time quantitative PCR (qPCR) (Additional file 2: Table S2). For that, 1.4 × 10^5^ cells were seeded in a 60 mm culture dish and cultured under N or AH for 48 h. After this time, total RNA was extracted using the mirVana™ miRNA Isolation kit (Invitrogen). The quality and the concentration of RNA were evaluated by Implen NanoPhotometer® (Thermo Fisher). cDNA was synthesized using iScript Reverse Transcription Supermix (BioRad, CA, USA), and the amplification was performed with commercial TaqMan® Fast Advanced Master Mix and TaqMan® Gene Expression Array Plates in combination with TaqMan® Gene Expression Assays (Thermo Fisher). All procedures were made following the protocol provided by the manufacturer. Data from each TaqMan Assays were acquired by QuantStudio 3 Real-Time PCR System (Applied Biosystems, Thermo Fisher Scientific, MA, USA) and analyzed with Thermo Fisher Cloud software using the 2^−ΔΔCt^ method. *GAPDH* was used as a reference gene. All assays were performed in duplicates.

Furthermore, gene expressions were represented in a principal component analysis (PCA) and a heat map, using ClustVis 2.0 (https://biit.cs.ut.ee/clustvis/). Functional enrichment analysis was performed using FunRich Version 3.1.4 (http://www.funrich.org/) to determine the gene ontology (GO) categories enriched or depleted with respect to the N condition. Finally, interaction network analysis of significantly expressed genes and the analysis of representative GO categories were performed with OmicsBeans (http://www.omicsbean.cn) and visually represented with Cytoscape 3.8.2.

### Isolation of secretome derived from MenSCs 

For secretome collection, MenSCs (n = 5) at passages P6–P9 were cultured in DMEM with 1% penicillin/streptomycin, 1% glutamine, and 1% insulin-transferrin-selenium (ITS, Thermo Fisher Scientific) for 48 h under N (N/S-MenSCs) and AH (AH/S-MenSCs) conditions. ITS replaced FBS to avoid external EV contamination. After that, conditioned media were collected and centrifuged, first at 1000×*g* for 10 min at 4 ºC and then at 5000×*g* for 20 min at 4 ºC to eliminate debris and dead cells. The supernatants were filtered through 0.45 µm and 0.22 µm meshes (Fisher Scientific, Leicestershire, UK) and concentrated by centrifugation at 4000×*g* for 40 min at 4 °C, using a 3 kDa MWCO Amicon® Ultra device (Merck-Millipore, MA, USA). Finally, protein concentration was measured by Bradford assay (Bio-Rad). The concentrated secretomes were stored at – 80 °C for further analyses.

### miRNA expression in secretomes from MenSCs

To examine the miRNA composition of the secretome derived from MenSCs under N and AH, the expression of 46 miRNAs was studied by qPCR (Additional file 3: Table S3). Total RNA from the secretome was extracted using Total Exosome RNA and Protein Isolation Kit, quantified with Implen NanoPhotometer® (Thermo Fisher), and retrotranscribed to cDNA using TaqMan® Advanced miRNA cDNA Synthesis kit (Thermo Fisher). cDNA amplification was performed with commercial TaqMan™ Fast Advanced Master Mix and designed TaqMan® Advanced miRNA Human Array plates together with TaqMan™ Advanced miRNA Assays (Thermo Fisher) and carried out in a QuantStudio 3 Real-Time PCR System (Applied Biosystems). The qPCR products were analyzed with Thermo Fisher Cloud software using the 2^−ΔΔCt^ method and normalized with hsa-let-7d-5p, after testing its stability. Moreover, a gene-target analysis was performed on the two significant miRNAs using mirTargetlink 2.0. The association of the strongly validated genes with the GO categories of interest was evaluated with OmicsBean (http://www.omicsbean.cn/) and, finally, an interaction network was created and visually represented with Cytoscape 3.8.2.

### Functional assays

#### Culture of HUVECs

For routine culture, HUVECs were seeded on 0.1% gelatin-coated (Sigma-Aldrich, MO, USA) T75 flasks and cultured in endothelial cell basal medium (EBM-2, Lonza, Basel, Switzerland) enriched with growth factors (EGM-2, SingleQuots™, Lonza). For the following described experiments, positive controls were set up with EGM-2 medium (supplemented EBM-2) while negative controls or starving conditions were set up with M199 + 1% FBS, N/S-MenSC, and AH/S-MenSCs. EGM-2 medium is the recommended medium for optimal the culture of endothelial cells such as HUVECs. On the other hand, it is necessary to perform the assays with a non-enriched culture medium with low FBS concentration to minimize possible external effects on our treatment. For that, HUVECs treatments with secretomes from MenSC (N and AH conditions) were performed under starving conditions (M199 + 1%FBS). HUVECs were used at maximum passage number 5. Three independent experiments were performed for each assay.

#### HUVEC proliferation assay

HUVECs were seeded at the density of 1 × 10^4^ cells per well on 0.1% gelatin-coated 96-well plates and incubated for 24 h to allow cell adhesion. HUVECs, under starving conditions, were treated with secretome derived from different MenSC culture conditions (N and AH), at a concentration of 100 µg/ml for 24 h. Cell proliferation was measured by CCK-8 assay according to the manufacturer’s instructions. Images of the wells were taken using an inverted microscope (Nikon Elipse TE2000-S) and analyzed using ImageJ (Schneider et al. 2012).

#### HUVEC migration assay

HUVEC migration was examined using a Boyden chamber of 6.5 mm polycarbonate membrane with 5 μm pores (Costar, Corning, NY, USA). HUVEC suspensions (5 × 10^4^ cells in 100 μl of M199 + 1% FBS) were added to the upper compartment. The bottom chambers of Transwell were filled with secretome derived from MenSCs under N or AH conditions at 100 μg/ml concentration. After 24 h, cells on the upper side of the membrane (non-migrated cells) were scraped with a cotton ball, and cells spreading on the bottom side of the membrane (invasive cells) were fixed with methanol (Cromakit, Granada, Spain) and stained with eosin-thiazine. Images were taken by inverted microscope (Nikon Elipse TE2000-S) and analyzed using ImageJ with Cell Counter plug-in.

#### HUVEC wound healing assay

Cell migration ability was also quantified using a scratch wound-healing assay. An artificial wound was created in HUVECs on an 80–90% confluent cell monolayer in a 24-well plate using a 200 μl pipette tip. Immediately after, 5 μg/ml mitomycin C (Sigma-Aldrich, MO, USA) was added to the culture wells to eliminate the influence of cell proliferation. The effects on HUVEC migration of co-cultured with secretomes from MenSCs under N or AH at 100 μg/ml concentration were monitored by microscopy at 0, 6, and 24 h. Images were taken using an inverted microscope and analyzed by ImageJ. The number of migrated cells was counted in the wounding zone determined by a predefined frame.

#### HUVEC tube formation assay

HUVEC tube formation capacity was analyzed by using an Angiogenesis μ-slide system (IBIDI GmbH, Planegg/Martinsried, Germany). μ-slide wells were coated with 10 μl growth factor reduced (GFR) Matrigel (BD Biosciences) for at least 30 min at 37 °C. After matrigel polymerization, HUVECs at a density of 2 × 104 were plated and incubated at 37 ºC for 24 h in the presence of secretomes from MenSCs under N or AH conditions at 100 μg/ml concentration. Images were taken with an inverted microscope (Nikon Elipse TE2000-S) and analyzed by using Image J Software with an Angiogenesis Analyzer plug-in.

### Statistical analysis

Data were statistically analyzed with GraphPad Prism (version 8.0). For analysis of differences between the two groups, Student's *t-*test was performed, and One-way ANOVA and Tukey’s multiple comparisons test were used for multiple groups. Data are presented as mean ± SD considering at least three independent replicates for each assay. The *p* values ≤ 0.05 were considered statistically significant. In all cases: *p < 0.05, **p < 0.005, and ***p < 0.0005.

## Results

### Phenotypic markers modification by acute hypoxia culture

**Fig. 1:**
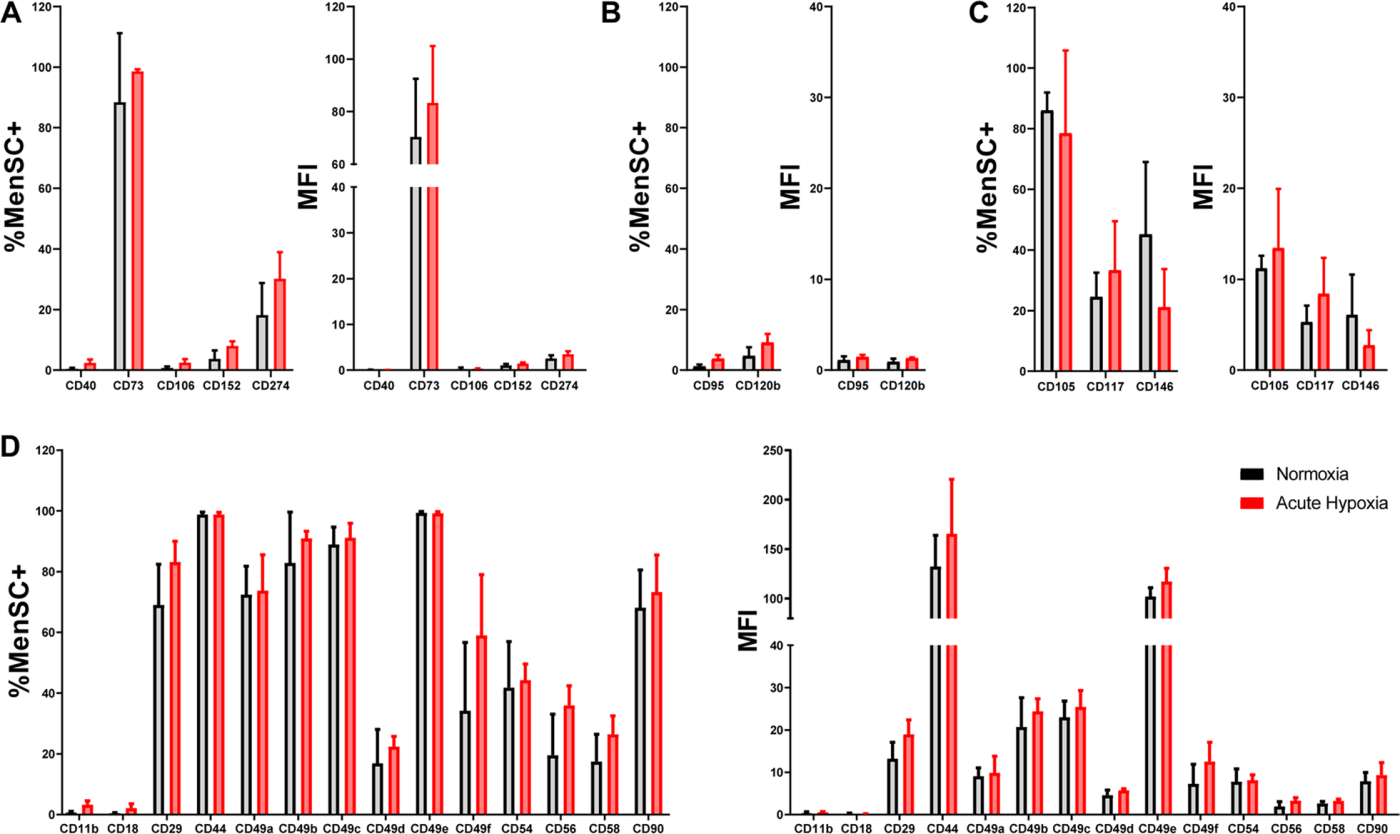
Expression of phenotypic surface markers on MenSCs. Analysis of surface markers of MenSCs under acute hypoxia (red bars, n = 5) condition. Graph bars show the percentage of positive MenSCs and the mean fluorescence intensity (MFI) of the different surface markers **A** immune-related markers, **B** apoptosis-related markers, **C** angiogenesis-related markers, and **D** cell adhesion/migration-related markers. A paired t-test was performed to compare acute hypoxia with normoxia. A p < 0.05 was considered statistically significant

Surface marker analysis was performed under the different preset oxygen concentrations (20.9%—Normoxia [N] and 0.1–1%—Acute Hypoxia [AH]). The data showed the % positive MenSCs and mean fluorescence intensity (MFI) of both biogroups (N and AH). No significant differences were detected in any marker. Immune-related markers showed a non-significant increase of CD73, CD152, and CD274 in both % MenSCs^+^ and MFI, for CD40 and CD106 hardly any expression was detected (Fig. 1A). The surface markers, CD95 and CD120b (apoptosis-related markers) showed a non-significant upward trend under AH (Fig. 1B). In the case of markers associated with angiogenesis, in terms of % MenSC^+^ CD105 and CD146 decrease with HA, while CD117 increases and in terms of MFI CD105 and CD117 increase, and CD146 confirms its decreasing trend with HA (Fig. 1C). As for cell adhesion and migration, both % MenSC^+^ and MFI increase for all markers non-significantly.

### Effects of hypoxia on proliferation and viability

**Fig. 2:**
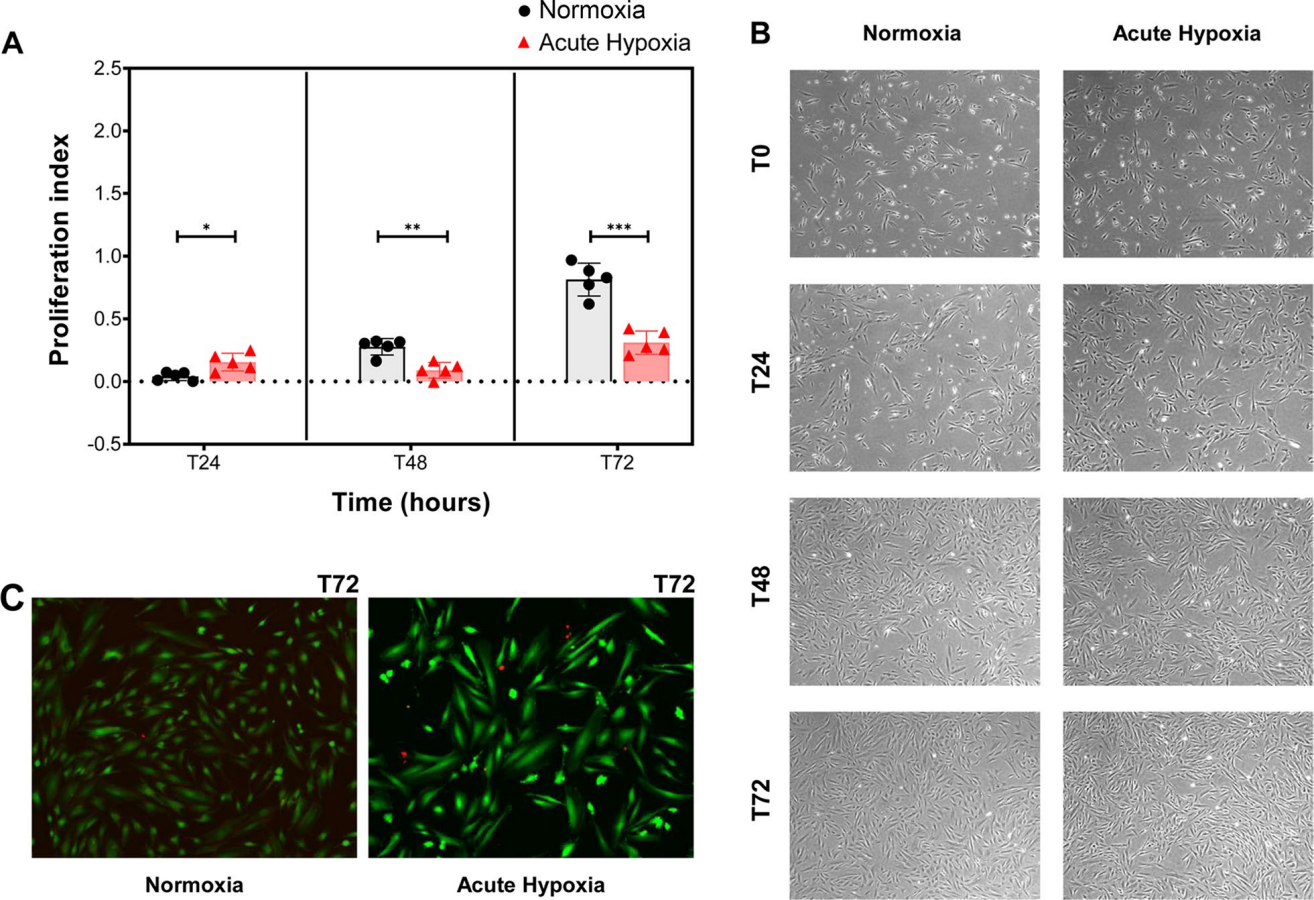
Effects of acute hypoxia on MenSCs: proliferation and viability. **A** Proliferation assay in MenSCs under normoxia (black dots, n = 5) or in acute hypoxia (red triangles, n = 5) at 24, 48, and 72 h. Values were normalized to the results at time 0 h. A paired t-test was performed and p < 0.05 was considered statistically significant. Error bars represent the standard deviations of data. Asterisks indicate statistically significant differences: *p < .05, **p < .005, *** p < .0005. **B** Optical microscopy images of proliferation in the culture flask under normoxia and acute hypoxia conditions, respectively, at 4x. **C** Representative images of the LIVE/DEAD viability assay at 10 × and 72 h. Red cells are considered dead cells

MenSC proliferation rate was quantified by CCK-8 assay and normalized to the baseline values of the cells at the beginning of the experiment. The assessment of MenSC proliferation under AH revealed a significant increase in their proliferation at 24 h, but after this time, a significant decrease was observed (Fig. 2A). These data correlated with microscopy follow-up (Fig. 2B). In the Live/Dead assays at 72 h, no viability changes were detected between different culture conditions (Fig. 2C).

### MenSCs gene expression

**Fig. 3:**
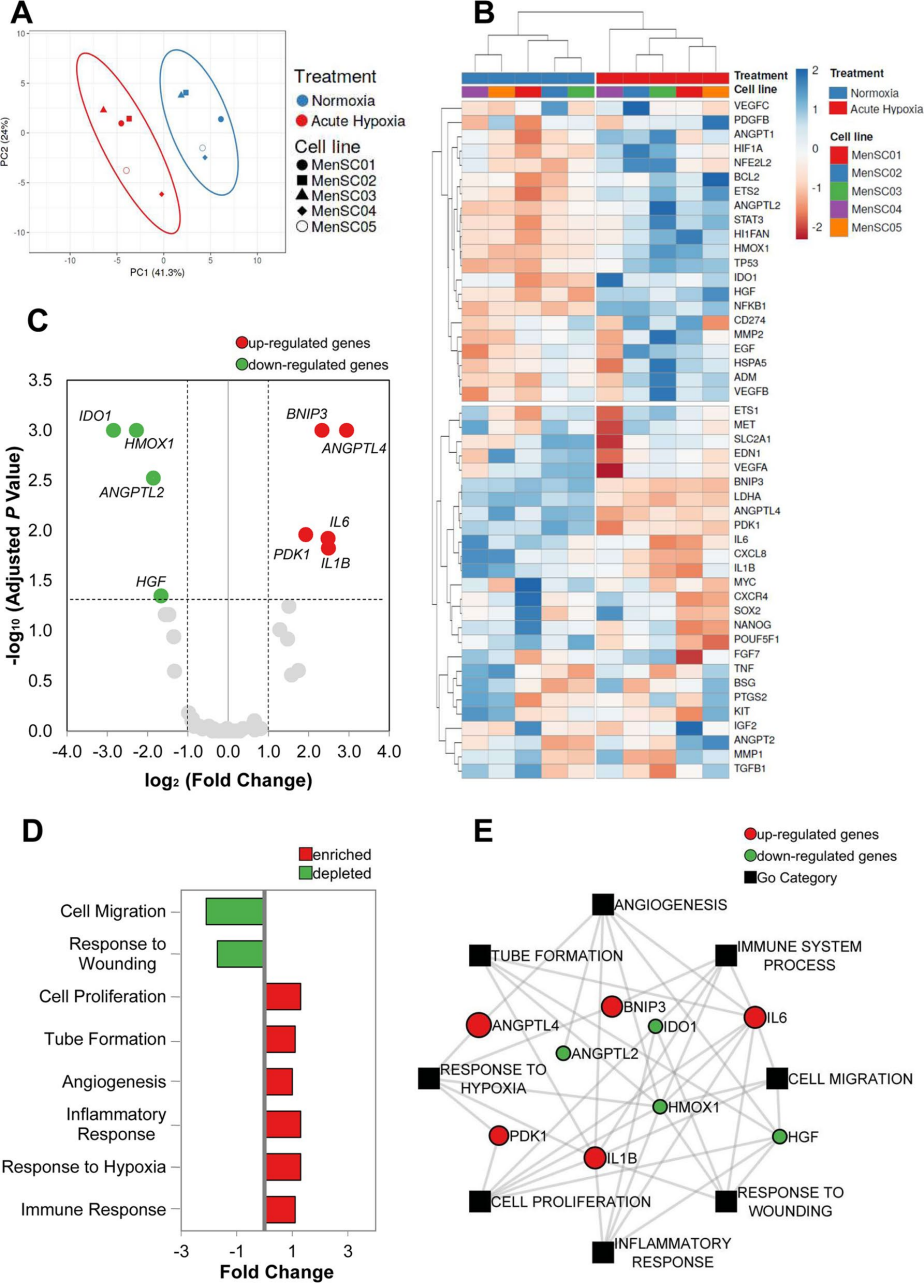
MenSCs Gene analysis. **A** Principal Component Analysis (PCA) plots (normoxia in blue and acute hypoxia in red). Score plots for PC1 and PC2 explain 41.3% and 24% of the total variance, respectively. **B** Hierarchical Clustering of the cell lines (MenSC01, MenSC02, MenSC03, MenSC04, and MenSC05) and the different conditions (normoxia in blue and acute hypoxia in red). **C** Volcano plot of differentially expressed genes (DEGs) identified between normoxia and acute hypoxia conditions. The red dots denote up-regulated gene expression, and the green dots denote down-regulated gene expression. **D** Quantitative enrichment analysis with the expression of all genes studied (enriched in red, depleted in green). **E** Cytoscape-designed interaction network including DEGs and GO categories of interest (up-regulated genes in red and down-regulated genes in green, categories are represented with a black square)

To further evaluate the effects of acute hypoxia, the expression of 60 genes was analyzed by qPCR. It was not possible to detect expression in 13 of them. The PCA analysis identified two well-differentiated groups (Fig. 3A). Likewise, the clustering analysis, represented as a heatmap, highlighted a differential expression between N and AH conditions (Fig. 3B). Lastly, differentially expressed genes (DEGs) were visualized in a Volcano plot. A total of four DEGs were significantly down-regulated in MenSCs under AH: *ANGPTL2* (*p* = 0.003), *HGF* (*p* = 0.045), *HMOX1* (*p* = 0.001), and *IDO1* (*p* = 0.001); while five DEGs were up-regulated: *ANGPTL4* (*p* = 0.001), *BNIP3* (*p* = 0.001), *IL6* (*p* = 0.012), *PDK1* (*p* = 0.011), and *IL1B* (*p* = 0.015) (Fig. 3C).

Gene expression data from both culture conditions were used to determine if the selected biological processes were enriched or depleted in AH compared with N (Fig. 3D). This analysis revealed that angiogenesis, tube formation, response to hypoxia, proliferation, and inflammatory and immune response GO categories were enhanced in AH, while other processes related to cell migration and response to wounding were reduced. Additionally, an interaction network was created to show the relation between DEGs and these GO categories (Fig. 3E).

### Analysis of miRNAs in the secretome of MenSCs

**Fig. 4:**
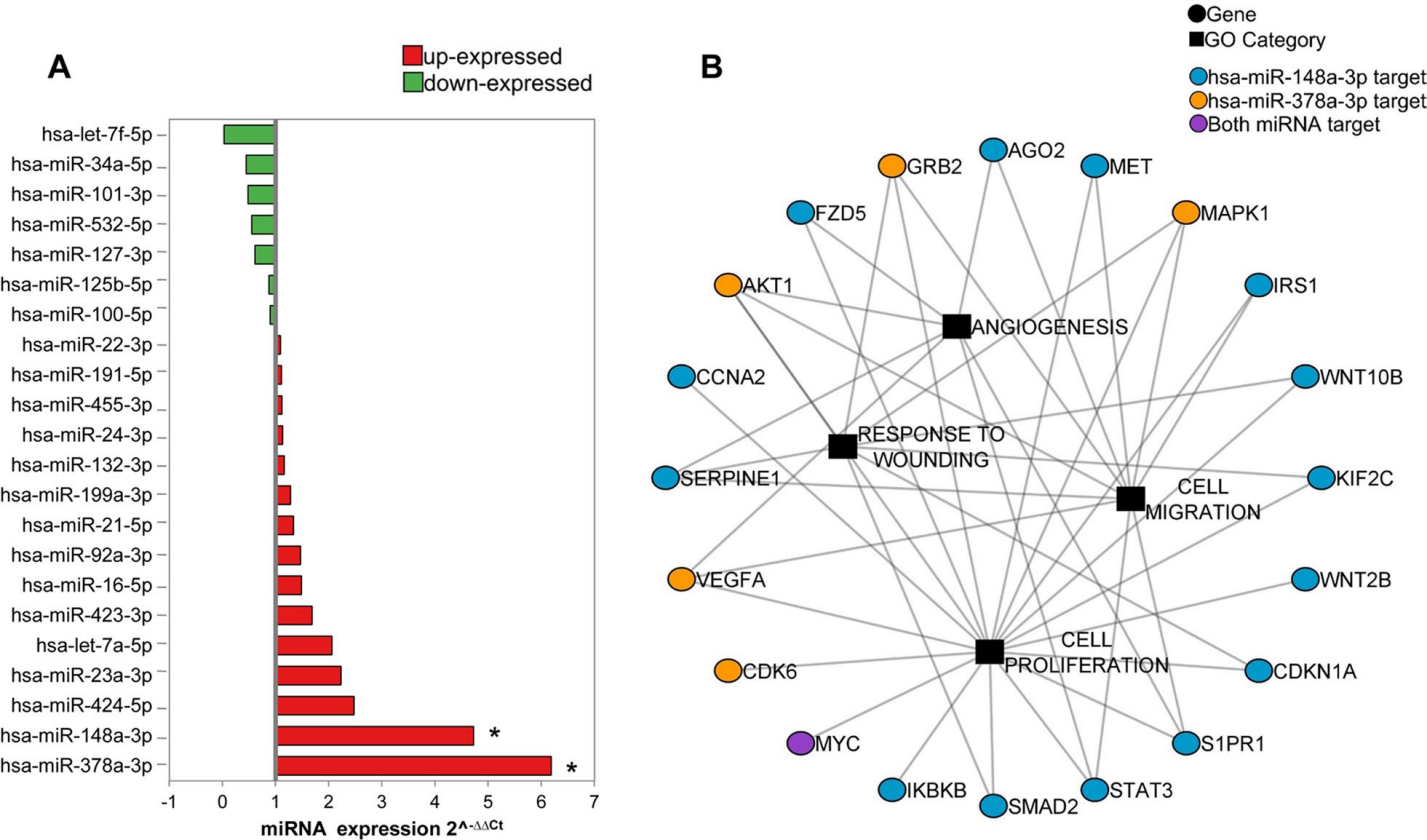
Analysis of the secretome miRNA cargo. Comparative analysis of miRNA expression in acute hypoxia in relation to normoxia. **A** The comparative analysis of miRNA expression is represented by the 2-ΔΔCt (up-expressed miRNA in red and down-regulated miRNAs in green). **B** Interaction network between the target genes of the significantly differentially expressed miRNA and the GO categories to which they are related. Genes are represented by a circle and GO categories by a square. The target genes of hsa-miR-148A-3p are in blue dots and those of hsa-miR-378A-3p are in yellow dots. The only common target gene is indicated in purple

The analysis of miRNAs in the secretomes from MenSCs under N or AH revealed a detectable expression of 22 out of 46 studied miRNAs, being miR-148a-3p, hsa-miR-378a-3p, hsa-miR-424-5p, hsa-miR-23a-3p, and hsa-miR-let-7a-5p the most widely expressed miRNAs in AH and hsa-miR-34a-5p in N. Three of them were only expressed under the N condition (hsa-miR-532-5p, hsa-miR-221-3p, hsa-miR-93-5p). Most miRNAs were found to be up-regulated (68%) in AH regarding N condition, including hsa-miR-148a-3p and hsa-miR-378a-3p which were statistically significant (Fig. 4A). The gene-target analysis of these miRNAs and their relationship with GO categories was analyzed in an interactions network (Fig. 4B).

### Functional assays

**Fig. 5:**
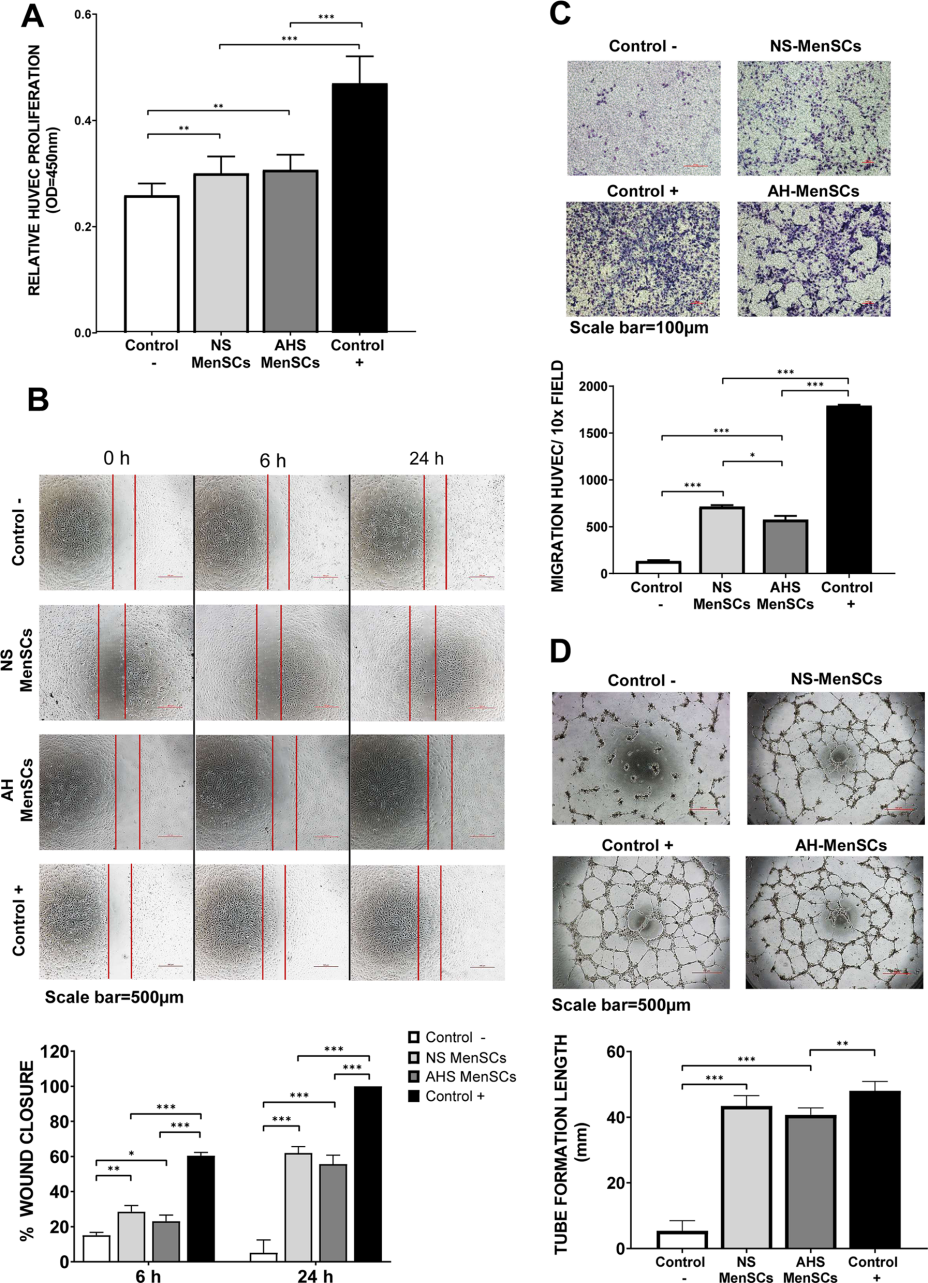
Functional assays on HUVECs. The effect of MenSC secretomes in the different conditions of the study (Normoxia [N] and Acute Hypoxia [AH]) on HUVEC cells. For the whole figure: negative control (Control -) in white, the treatment with secretome derived from normoxic MenSCs (N/S-MenSCs) in light grey, the treatment with secretome derived from acute hypoxic MenSCs (AH/S-MenSCs) in dark grey, and positive control (Control +) in black. Control -: M199 medium + 1% FBS (starving condition). N/S- and AH/S-MenSCs: M199 medium + 1% FBS + secretome treatment (100 µg/ml). Control + : EGM-2 medium. **A** Relative proliferation of HUVECs during 24 h with respect to their initial confluence. **B** Percentage of wound closure. **C** Migration of HUVECs. **D** Length of the tube formed in mm. Analysis: One-way ANOVA and Tukey’s multiple comparisons tests. Bar graphs show average values, error bars: SEM. Asterisks indicate statistically significant differences: *p < .05, **p < .005, *** p < .0005

HUVECs were exposed to MenSCs secretomes to evaluate whether they could exert an effect on endothelial cell proliferation, migration, and angiogenic capacity. HUVEC proliferation was quantified in a CCK-8 assay. MenSCs secretome from both oxygen conditions (N and AH) significantly increased the HUVEC proliferation compared with the negative control (Fig. 5A). In addition, HUVEC migration was determined in a wound-healing assay (Fig. 5B) and by transwell assays (Fig. 5C). In both assays, the secretomes from MenSCs increased HUVEC migration compared to the negative control. Finally, the angiogenic activity of secretomes was in vitro evaluated through tube formation assay. The secretomes were able to induce HUVECs to form tube-like structures in vitro (Fig. 5D). A significant increase in the total length of tubes was identified in those HUVECs co-cultured with the MenSCs secretomes.

## Discussion

Despite the tremendous effort to overcome the current limitations of tissue regeneration therapies, the treatment of chronic wounds remains ineffective. Up to now, one of the most promising approaches is the use of MSCs as an alternative therapy. It is widely accepted that in vitro MSCs can recruit endothelial cells, improving their proliferation, migration, and angiogenesis as part of tissue repair mechanisms (Chen and Huang 2018), being the most studied sources: bone marrow-derived MSCs (BM-MSCs), adipose-derived MSCs (ADSCs), and umbilical cord-derived MSCs (UC-MSCs) (Cuenca et al. 2018). Unfortunately, their efficacy in vivo is limited by their poor survival rate after transplantation due to the hostility of the wound micro-environment (Sylakowski et al. 2020). Several attempts have been made to culture MSCs at similarly low oxygen levels to condition or acclimate them prior to implantation in the wound with limited success (Sylakowski et al. 2020). When MSCs are exposed to acute hypoxia [< 1.5% O_2_), as occurs in the wound bed after transplantation, they undergo a massive increase in apoptosis (Sylakowski et al. 2020; Khasawneh et al. 2019). In this context, the development of new strategies to improve MSC survival and efficacy becomes a priority. Therefore, to our knowledge, this is the first work focused on the evaluation of the therapeutic potential of MenSCs under acute hypoxia condition.

As expected, our findings after the gene expression analysis confirmed that hypoxic preconditioning up-regulates genes associated with energy metabolism (*PDK1*, *SLC2A1*, and *LDH*). This may be attributed to a shift from aerobic respiration through oxidative phosphorylation to anaerobic respiration through glycolysis (Antebi et al. 2018). Moreover, under hypoxia conditions there is a characteristic up-regulation of multipotency (Samal et al. 2021), this undifferentiated state can be observed in our data in the over-expression of the pluripotent genes *NANOG*, *POUF5F1*, and *SOX2*. Interestingly, the expression of *HIF1A* and *NKFB*, both key molecules for the regulation of gene expression during the hypoxic response, decreases after prolonged exposure of cells to hypoxic conditions (Nakayama and Kataoka 2019), which agrees with our results. All these results support that MenSCs were subjected to low 0_2_ concentrations.

It is known that wound healing involves biological processes such as inflammation, proliferation, and remodeling. In this context, inflammation is one of the major challenges in wound treatment. In this regard, it is interesting to note that we have previously demonstrated that secretomes of TNFα/IFNγ-primed MenSCs had an immunomodulatory potential on immune cells (Pedro et al. 2021). This capacity seems to be maintained also with AH preconditioning, according to our data, the GO categories: *Immune response* (GO:0006955), and *Inflammatory response* (GO:0006954) were also increased compared with basal cells in normoxia.

Furthermore, our in vitro findings demonstrate that, under acute hypoxia, the viability of MenSCs is not affected, although their proliferation is partially reduced. These observations can be justified by our results of the down-regulation of apoptotic genes such as BCL2 or TP53 and the significant increase of BNIP3 expression, which is associated with a pro-survival phenotype (Filippi et al. 2018). On the contrary, we have also detected down-regulated HMOX1 expression that is associated with elevated cellular stress and cell mortality (Leung et al. 2017). The exact mechanism by which MenSCs tolerate acute hypoxia well is still unclear and needs to be further investigated. However, it could be related to the role of hypoxia during physiological menstruation in driving the repair process and limiting menstrual blood loss (Reavey et al. 2021).

The remodeling capacity, in turn, involves the processes of migration, angiogenesis, and wound healing. MenSCs have been shown to have a superior ability to migrate to the site of injury in response to cell damage signals than other sources of MSCs (Cuenca et al. 2018). HGF is one of the factors responsible for the regulation of target cell migration through the induction of the HGF/cMET pathway (Leung et al. 2017). Our data showed significant downregulation of HGF expression. It may be the cause of the migration reduction compared to normoxic MenSCs observed with bioinformatic analyses. Moreover, the increased angiogenesis also observed in silico may be mainly due to the upregulated expression of three proinflammatory cytokines: IL6, involved in angiogenic processes and endothelial cell proliferation (Cuenca et al. 2018); IL1B, which acts regulating vascular permeability and angiogenesis (Fahey and Doyle 2019), and ANGPTL4, which is associated with the regulation of vascular integrity (Carbone et al. 2018). On the other hand, the decreased wound closure category in MenSCs under acute hypoxia may be mediated by decreased expression of ANGPTL2. This pro-angiogenic factor positively regulates vasculogenesis by promoting cell migration and contributes to tissue repair by inducing tissue remodeling in coordination with MMPs activity (Thorin-Trescases et al. 2021). Considering this, AH seems to affect the remodeling capacity of MenSCs, but not in a dramatic way as demonstrated by the results of the assays on endothelial cells discussed below.

The paracrine effects of MenSCs can be also mediated by the miRNAs released in secretome samples. Here we show that hsa-miR-148a-3p was significantly up-regulated under acute hypoxia, this miRNA has been found to promote proliferation and angiogenesis in vascular endothelial cells (Wang et al. 2018). Moreover, it is found to be involved in regulating transcripts related to cell cycle, focal adhesion, VEGF signaling, and wound healing mechanism. In addition, this miRNA is likely to be involved in NF-κB signaling, and inflammatory gene expression (Kasiviswanathan et al. 2020). The other significantly up-regulated miRNA, hsa-miR-378a-3p, is implicated in metabolic pathways, mitochondrial energy homeostasis, and related biological processes such as muscle development, differentiation, and regeneration (Krist et al. 2015). In addition, it has been related to the modulation of proangiogenic factors such as vascular endothelial growth factor, angiopoietin-1, or interleukin-8, it influences inflammatory response and affects tumor suppressors (Krist et al. 2015). After the analysis of target genes, *MYC* turned out to be the only target gene common to both miRNAs. This transcription factor is involved in multiple processes such as the regulation of growth, differentiation, and cell death, in addition to inducing the expression of genes involved in angiogenesis. *MYC* is a positive regulator of HIF and its activity is required for the normal hypoxic response and hypoxia-dependent glycolytic reprogramming mentioned above (Nakayama and Kataoka 2019). Altogether, the miRNA analyses in the secretomes from MenSCs under normoxia and acute hypoxia suggest that they may be involved in part in the beneficial effects observed in the in vitro assays.

Lastly, our in vitro assays performed with endothelial cells (HUVECs) have demonstrated that secretomes from MenSCs, under both oxygen concentrations (N and AH), were able to increase proliferation, migration, wound repair, and tube formation indicating that the biological functions of MenSCs are almost not altered using this priming strategy. These studies also corroborate what was observed at the gene level, AH partially limits the in vitro effectiveness of MenSCs in terms of migration and wound closure.

In this study, we have demonstrated that the source of the cells is one of the main factors in the therapy election. MenSCs have been shown not only to be able to proliferate and survive in a low-oxygen environment but also to maintain their therapeutic potential and could be applied in the treatment of pathologies associated with these environments, as their secretome has been shown to maintain their excellent properties. However, in vivo studies must be performed to confirm these findings.

## Conclusion

In conclusion, this study provides insight into the therapeutic capacity of the secretome of MenSCs under acute hypoxia and postulates its use as a promising therapeutic approach in pathologies with low oxygen environments such as chronic wounds.

## Supplementary Information

**Additional file 1: Table S1.** Panel used for the MenSCs phenotypic characterization.

**Additional file 2: Table S2.** Assays ID of commercial TaqMan gene Assays.

**Additional file 3: Table S3.** Assays ID of commercial TaqMan miRNA Assays.

## Data Availability

Not applicable.

## Abbreviations

AH: Acute hypoxia
AH/S-MenSCs: Secretome from menstrual blood-derived stromal cells under acute hypoxia
CCK-8: Cell Counting Kit 8
DEG: Differential expressed genes
DMEM: Dulbecco’s Modified Eagle’s medium
EBM-2: Endothelial cell basal medium
EGM-2: Endothelial cell basal medium enriched with growth factors
EVs: Extracellular vesicles
FBS: Fetal bovine serum
GFR: Growth factor reduced
GO: Gene ontology
HUVECs: Human umbilical vein endothelial cells
ISCT: International Society for Cell Therapy
ITS: Insulin-transferrin-selenium
MenSCs: Menstrual blood-derived stromal cells
MSCs: Mesenchymal stromal cells
MFI: Mean fluorescence intensity
N: Normoxia
N/S-MenSCs: Secretome from menstrual blood-derived stromal cells under normoxia
qPCR: Real-time quantitative PCR
PCA: Principal component analysis
