# Supplementary material for: Menstrual blood-derived stromal cells: insights into their secretome in acute hypoxia conditions
Source: Mol Med. 2023 Apr 4;29:48. doi: 10.1186/s10020-023-00646-1 (PMC10074862; doi:10.1186/s10020-023-00646-1)
Supplement: Supplementary file 1 — Additional file 1: Table S1. Panel used for the MenSCs phenotypic characterization. [file 10020_2023_646_MOESM1_ESM.docx]

Table S1

| **Antigen** | **Clone** | **Catalog number (Company)** |
| --- | --- | --- |
| **CD11b** | ICRF44 | MCA551F (Bio-Rad) |
| **CD14** | MEM-18 | MCA2185 (Bio-Rad) |
| **CD18** | 6.7. | 555923 (BD Pharmingen) |
| **CD29** | 4B7R | MCA1949F (Bio-Rad) |
| **CD34** | QBEND/10 | MCA547F (Bio-Rad) |
| **CD40** | 5C3 | 555589 (BD Pharmingen) |
| **CD44** | 515 | 550989 (BD Pharmingen) |
| **CD45** | F10-89-4 | MCA87F (Bio-Rad) |
| **CD49a** | SR84 | 559596 (BD Pharmingen) |
| **CD49b** | AK7 | MCA743F (Bio-Rad) |
| **CD49c** | 17C6 | MCA1948F (Bio-Rad) |
| **CD49d** | 44H6 | MCA923F (Bio-Rad) |
| **CD49e** | JBS5 | MCA1187T (Bio-Rad) |
| **CD49f** | 450-30A | MCA1457F (Bio-Rad) |
| **CD54** | 15.2 | MCA1615F (Bio-Rad) |
| **CD56** | B159 | 555516 (BD Pharmingen) |
| **CD58** | B-L28 | 854.632.010 (DIACLONE) |
| **CD73** | AD2 | 550257 (BD Pharmingen) |
| **CD90** | F15-42-1 | MCA90F (Bio-Rad) |
| **CD95** | LOB 3/17 | MCA1539F (Bio-Rad) |
| **CD105** | SN6 | MCA1557 (Bio-Rad) |
| **CD106** | 51-10C9 | 551146 (BD Pharmingen) |
| **CD107a** | H4A3 | A15798 (Thermo Fisher) |
| **CD117** | A3C6E2 | 130-091-733 (MACS) |
| **CD120b** | MR2-1 | MCA1944 (Bio-Rad) |
| **CD126** | M5 | 551850 (BD Pharmingen) |
| **CD133** | 293C3 | 130-090-853 (Miltenyi Biotec) |
| **CD146** | P1H12 | 562135 (BD Pharmingen) |
| **CD152** | BNI3 | 555853 (BD Pharmingen) |
| **CD166** | 3A6 | MCA1926F (Bio-Rad) |
| **CD274** | MIH1 | 12-5589-73 (eBioscience) |
| **CD279** | MIH4 | 558694 (BD Pharmingen) |
| **HLA-ABC** | G46-2.6 | 555553 (BD Pharmingen) |
| **HLAII DR** | WR18 | MCA477PE (Bio-Rad) |
