# Supplementary material for: Menstrual blood-derived stromal cells: insights into their secretome in acute hypoxia conditions
Source: Mol Med. 2023 Apr 4;29:48. doi: 10.1186/s10020-023-00646-1 (PMC10074862; doi:10.1186/s10020-023-00646-1)
Supplement: Supplementary file 2 — Additional file 2: Table S2. Assays ID of commercial TaqMan gene Assays. [file 10020_2023_646_MOESM2_ESM.docx]

Table S2

| **Gene** | **Assay ID/Name** | **Gene** | **Assay ID/Name** |
| --- | --- | --- | --- |
| **18S** | Hs99999901_s1 | **IGF2** | Hs01005963_m1 |
| **ADM** | Hs00181605_m1 | **IL10** | Hs00961622_m1 |
| **ANGPT1** | Hs00181613_m1 | **IL1B** | Hs01555410_m1 |
| **ANGPT2** | Hs00169867_m1 | **IL6** | Hs00174131_m1 |
| **ANGPT4** | Hs00211115_m1 | **IL8** | Hs00174103_m1 |
| **ANGPTL2** | Hs00765775_m1 | **KIT** | Hs00174029_m1 |
| **ANGPTL4** | Hs01101127_m1 | **LDHA** | Hs00855332_g1 |
| **ARG1** | Hs00163660_m1 | **MMP1** | Hs00899658_m1 |
| **BCL2** | Hs00608023_m1 | **MMP2** | Hs00234422_m1 |
| **BNIP3** | Hs00969291_m1 | **MMP9** | Hs00957562_m1 |
| **BSG** | Hs00174305_m1 | **MYC** | Hs00153408_m1 |
| **c-MET** | Hs01565584_m1 | **NANOG** | Hs02387400_g1 |
| **CTLA4** | Hs00175480_m1 | **NFE2L2** | Hs00975961_g1 |
| **CXCR4** | Hs00607978_s1 | **NFKB1** | Hs00765730_m1 |
| **EDN1** | Hs00174961_m1 | **NOS2** | Hs01075529_m1 |
| **EGF** | Hs01099999_m1 | **PDGFB** | Hs00234042_m1 |
| **ETS1** | Hs00428293_m1 | **PDK1** | Hs01561850_m1 |
| **ETS2** | Hs00232009_m1 | **PDL1** | Hs00204257_m1 |
| **FGF7** | Hs00940253_m1 | **POUF5F1** | Hs04260367_gh |
| **GAPDH** | Hs99999905_m1 | **PTGS2** | Hs00153133_m1 |
| **GUSB** | Hs99999908_m1 | **SLC2A1** | Hs00892681_m1 |
| **HGF** | Hs00300159_m1 | **SOX2** | Hs01053049_s1 |
| **HIF1A** | Hs00153153_m1 | **STAT3** | Hs00374280_m1 |
| **HIF1AN** | Hs00215495_m1 | **TGFA** | Hs00608187_m1 |
| **HIF3A** | Hs00541709_m1 | **TGFB1** | Hs00998133_m1 |
| **HMOX1** | Hs00157965_m1 | **TNF** | Hs00174128_m1 |
| **HPRT1** | Hs99999909_m1 | **TP53** | Hs00153349_m1 |
| **HSPA5** | Hs99999174_m1 | **VEGFA** | Hs00900054_m1 |
| **IDO** | Hs00984148_m1 | **VEGFB** | Hs00173634_m1 |
| **IGF1** | Hs01547656_m1 | **VEGFC** | Hs00153458_m1 |
