# Supplementary material for: Menstrual blood-derived stromal cells: insights into their secretome in acute hypoxia conditions
Source: Mol Med. 2023 Apr 4;29:48. doi: 10.1186/s10020-023-00646-1 (PMC10074862; doi:10.1186/s10020-023-00646-1)
Supplement: Supplementary file 3 — Additional file 3: Table S3. Assays ID of commercial TaqMan miRNA Assays. [file 10020_2023_646_MOESM3_ESM.docx]

Table S3

| **miRNA** | **Assay ID/Name** | **miRNA** | **Assay ID/Name** |
| --- | --- | --- | --- |
| **let-7a-5p** | [478575_mir](https://www.thermofisher.com/order/genome-database/details/microrna/478575_mir?CID=&ICID=&subtype=) | **miR-15b** | [478313_mir](https://www.thermofisher.com/order/genome-database/details/microrna/478313_mir?CID=&ICID=&subtype=) |
| **let-7c** | [478577_mir](https://www.thermofisher.com/order/genome-database/details/microrna/478577_mir?CID=&ICID=&subtype=) | **miR-191-5p** | [477952_mir](https://www.thermofisher.com/order/genome-database/details/microrna/477952_mir?CID=&ICID=&subtype=) |
| **let-7d-3p** | [477848_mir](https://www.thermofisher.com/order/genome-database/details/microrna/477848_mir?CID=&ICID=&subtype=) | **miR-199a-3p** | [477961_mir](https://www.thermofisher.com/order/genome-database/details/microrna/477961_mir?CID=&ICID=&subtype=) |
| **let-7d-5p** | [478439_mir](https://www.thermofisher.com/order/genome-database/details/microrna/478439_mir?CID=&ICID=&subtype=) | **miR-208b** | [477806_mir](https://www.thermofisher.com/order/genome-database/details/microrna/477806_mir?CID=&ICID=&subtype=) |
| **let-7f** | [478578_mir](https://www.thermofisher.com/order/genome-database/details/microrna/478578_mir?CID=&ICID=&subtype=) | **miR-21-5p** | [477975_mir](https://www.thermofisher.com/order/genome-database/details/microrna/477975_mir?CID=&ICID=&subtype=) |
| **let-7i-3p** | [477862_mir](https://www.thermofisher.com/order/genome-database/details/microrna/477862_mir?CID=&ICID=&subtype=) | **miR-223** | [rno481007_mir](https://www.thermofisher.com/order/genome-database/details/microrna/rno481007_mir?CID=&ICID=&subtype=) |
| **miR-100-5p** | [478224_mir](https://www.thermofisher.com/order/genome-database/details/microrna/478224_mir?CID=&ICID=&subtype=) | **miR-22-3p** | [477985_mir](https://www.thermofisher.com/order/genome-database/details/microrna/477985_mir?CID=&ICID=&subtype=) |
| **miR-101** | [477863_mir](https://www.thermofisher.com/order/genome-database/details/microrna/477863_mir?CID=&ICID=&subtype=) | **miR-23a-3p** | [478532_mir](https://www.thermofisher.com/order/genome-database/details/microrna/478532_mir?CID=&ICID=&subtype=) |
| **miR-125b-5p** | [477885_mir](https://www.thermofisher.com/order/genome-database/details/microrna/477885_mir?CID=&ICID=&subtype=) | **miR-24** | [477992_mir](https://www.thermofisher.com/order/genome-database/details/microrna/477992_mir?CID=&ICID=&subtype=) |
| **miR-126-3p** | [477887_mir](https://www.thermofisher.com/order/genome-database/details/microrna/477887_mir?CID=&ICID=&subtype=) | **miR-29a-5p** | [478002_mir](https://www.thermofisher.com/order/genome-database/details/microrna/478002_mir?CID=&ICID=&subtype=) |
| **miR-126-5p** | [477888_mir](https://www.thermofisher.com/order/genome-database/details/microrna/477888_mir?CID=&ICID=&subtype=) | **miR-29b-3p** | [478369_mir](https://www.thermofisher.com/order/genome-database/details/microrna/478369_mir?CID=&ICID=&subtype=) |
| **miR-127-3p** | [477889_mir](https://www.thermofisher.com/order/genome-database/details/microrna/477889_mir?CID=&ICID=&subtype=) | **miR-29c-3p** | [479229_mir](https://www.thermofisher.com/order/genome-database/details/microrna/479229_mir?CID=&ICID=&subtype=) |
| **miR-130a-3p** | [477851_mir](https://www.thermofisher.com/order/genome-database/details/microrna/477851_mir?CID=&ICID=&subtype=) | **miR-34a** | [478048_mir](https://www.thermofisher.com/order/genome-database/details/microrna/478048_mir?CID=&ICID=&subtype=) |
| **miR-132** | [477900_mir](https://www.thermofisher.com/order/genome-database/details/microrna/477900_mir?CID=&ICID=&subtype=) | **miR-34c** | [478052_mir](https://www.thermofisher.com/order/genome-database/details/microrna/478052_mir?CID=&ICID=&subtype=) |
| **miR-133a** | [478706_mir](https://www.thermofisher.com/order/genome-database/details/microrna/478706_mir?CID=&ICID=&subtype=) | **miR-378** | [478349_mir](https://www.thermofisher.com/order/genome-database/details/microrna/478349_mir?CID=&ICID=&subtype=) |
| **miR-137** | [477904_mir](https://www.thermofisher.com/order/genome-database/details/microrna/477904_mir?CID=&ICID=&subtype=) | **miR-424-5p** | [478092_mir](https://www.thermofisher.com/order/genome-database/details/microrna/478092_mir?CID=&ICID=&subtype=) |
| **miR-139-3p** | [477906_mir](https://www.thermofisher.com/order/genome-database/details/microrna/477906_mir?CID=&ICID=&subtype=) | **miR-451a** | [478107_mir](https://www.thermofisher.com/order/genome-database/details/microrna/478107_mir?CID=&ICID=&subtype=) |
| **miR-142-5p** | [477911_mir](https://www.thermofisher.com/order/genome-database/details/microrna/477911_mir?CID=&ICID=&subtype=) | **miR-455-3p** | [478112_mir](https://www.thermofisher.com/order/genome-database/details/microrna/478112_mir?CID=&ICID=&subtype=) |
| **miR-145-5p** | [477915_mir](https://www.thermofisher.com/order/genome-database/details/microrna/477915_mir?CID=&ICID=&subtype=) | **miR-487b** | [478938_mir](https://www.thermofisher.com/order/genome-database/details/microrna/478938_mir?CID=&ICID=&subtype=) |
| **miR-146a** | [478399_mir](https://www.thermofisher.com/order/genome-database/details/microrna/478399_mir?CID=&ICID=&subtype=) | **miR-532-5p** | [478151_mir](https://www.thermofisher.com/order/genome-database/details/microrna/478151_mir?CID=&ICID=&subtype=) |
| **miR-148a-3p** | [477814_mir](https://www.thermofisher.com/order/genome-database/details/microrna/477814_mir?CID=&ICID=&subtype=) | **miR-92a** | [477827_mir](https://www.thermofisher.com/order/genome-database/details/microrna/477827_mir?CID=&ICID=&subtype=) |
| **miR-148a-5p** | [478718_mir](https://www.thermofisher.com/order/genome-database/details/microrna/478718_mir?CID=&ICID=&subtype=) | **miR-16** | [477860_mir](https://www.thermofisher.com/order/genome-database/details/microrna/477860_mir?CID=&ICID=&subtype=) |
| **miR-150** | [477918_mir](https://www.thermofisher.com/order/genome-database/details/microrna/477918_mir?CID=&ICID=&subtype=) | **miR-423** | [478327_mir](https://www.thermofisher.com/order/genome-database/details/microrna/478327_mir?CID=&ICID=&subtype=) |
